# Cognitive Stimulation with Music in Older Adults with Cognitive Impairment: A Scoping Review

**DOI:** 10.3390/brainsci14080842

**Published:** 2024-08-22

**Authors:** Alfredo Raglio, Camilla Figini, Alice Bencivenni, Federica Grossi, Federica Boschetti, Marina Rita Manera

**Affiliations:** 1 Music Therapy Research Laboratory, Istituti Clinici Scientifici Maugeri IRCCS, Via Maugeri, 27100 Pavia, Italy; 2 Independent Researcher, 22070 Locate Varesino, Italy; camilla196figini@gmail.com; 3 Associazione Raíces, 27100 Pavia, Italy; alice.bencivenni@gmail.com; 4 Psychology Unit Pavia-Montescano, Istituti Clinici Scientifici Maugeri IRCCS, 27100 Pavia, Italy; federica.grossi@icsmaugeri.it (F.G.); boschetti20@gmail.com (F.B.); marina.manera@icsmaugeri.it (M.R.M.)

**Keywords:** music-based interventions, music therapy, cognitive stimulation, cognitive training, cognitive impairment, mild cognitive impairment, dementia, cognitive outcomes

## Abstract

Background: The use of music in cognitive interventions represents a possibility with potential worthy of further investigation in the field of aging, both in terms of prevention from dementia, in the phase of mild cognitive impairment, and in the treatment of overt dementia. Objectives: Currently, the types of music-based interventions proposed in the literature are characterized by wide heterogeneity, which is why it is necessary to clarify which interventions present more evidence of effectiveness in stimulating different cognitive domains. Method: The study was conducted in accordance with PRISMA guidelines for scoping reviews. By searching two different databases, PubMed and the Web of Science, all studies evaluating the cognitive effects of music-based interventions on people at early stages of cognitive decline (MCI or mild-to-moderate dementia) were selected. Results: The study selection included a total of 28 studies involving n = 1612 participants (mean age ranged from 69.45 to 85.3 years old). Most of the studies analyzed agree with the observation of an improvement, or at least maintenance, of global cognitive conditions (mainly represented by the results of the MMSE test) following music-based interventions, together with a series of other positive effects on verbal fluency, memory, and executive processes. Conclusions: The results of this review suggest the introduction of music-based interventions as complementary approaches to usual cognitive treatments. Also, the use of standardized and well-defined protocols, in addition to strong methodological research approaches, is suggested. Music-based interventions are recommended in the early stages of dementia, in MCI, and in a preventive sense in healthy older adults.

## 1. Introduction

As the world’s population ages [1], the incidence of major neurocognitive disorders (NCDs), including Alzheimer’s disease (AD), is expected to increase in the coming years. The World Health Organization (WHO) has identified dementia as a global public health priority, with data from the WHO Global Action Plan 2017–2025 estimating that more than 55 million people worldwide are living with dementia, a number that is expected to rise to 75 million by 2030 and 132 million by 2050, with about 10 million new cases per year (1 every 3 s) [2]. Thus, dementia has a physical, psychological, social, and economic impact not only on those affected but also on their caregivers, families, and society, and it is a leading cause of disability and dependency among older adults worldwide [1].

Mild cognitive impairment (MCI), on the other hand, represents a borderline between physiological and pathological aging, characterized by a subjectively and objectively observed decline in one or more cognitive domains without interfering with the individual’s independence on daily activity performance [3,4].

The importance of a diagnosis of MCI lies primarily in the possibility of anticipating the progression to full-blown dementia, as patients with this diagnosis have an increased risk of developing Alzheimer’s disease, estimated at 1 to 25 percent per year [5]. This makes it possible to anticipate the implementation of cognitive interventions to increase the amount of protective cognitive resources.

In the literature, plasticity processes are reported in old age and also in neurodegenerative diseases, although to a lesser extent [6]; brain plasticity allows us to record brain reorganization corresponding to behavioral changes induced by cognitive interventions [7]. The neurodegenerative and progressive nature of dementia makes it difficult to restore impaired abilities through interventions, but they can maintain the current situation for as long as possible or reduce disease-related disability to some extent. The cognitive intervention that is best adapted to a mild to moderate level of dementia severity is cognitive rehabilitation, whose objectives focus on the difficulties that concretely affect the person’s functioning in daily life [8], following a compensatory approach.

Non-pharmacological treatments for dementia management recommended by the National Institute for Health and Care Excellence (NICE, 2018) include cognitive stimulation therapy (CST), an evidence-based protocol [9]. Multiple studies agree in supporting CST as an effective treatment to improve global cognitive function in patients with mild to moderate dementia [10,11,12]. For example, a multicenter controlled study by Carbone et al. [13] tested the efficacy of an Italian adaptation of the CST protocol developed by Spector and colleagues (CST-IT) [14] in a sample of people with mild to moderate dementia, both in the short term and at 3-month follow-up. The results show maintenance and even improvement in the performance of the experimental group on the MMSE and ADAS-Cog tests, compared with the active control group; these benefits were also confirmed in the long term [14]. It follows that CST can support cognitive function and counteract a person’s gradual cognitive decline (over a period of at least 3 months) [14]. Language performance also improved in the short term, and this benefit persisted at follow-up, highlighting that CST is particularly well suited for supporting communication skills in people with dementia [14].

Patients with MCI report a memory disturbance, which requires the individual to be aware of their own cognitive changes [14]. This characteristic, together with the preserved ability to learn new strategies, makes the MCI population particularly suited to benefit from cognitive training (CT) [14]. Episodic memory is the most severely impaired cognitive function and the main complaint in MCI [15]. For this reason, many cognitive training programs have focused on promoting and maintaining episodic memory in older adults with MCI. Memory training programs are typically based on teaching strategies that promote deeper encoding or facilitate retrieval and take advantage of aspects of memory that are relatively well preserved in MCI, such as semantic knowledge, visual imagery, or implicit retrieval [15]. Several studies have found these strategies to improve episodic memory outcomes, whether tested by immediate or delayed free recall of words [16,17], recognition [18], event-related prospective memory [19], or face–name associations [16]. Other cognitive domains include attention control and executive functions, skills highly involved in daily life whose impaired executive is predictive of disability in older adults. Very few studies, however, have focused on the training of these global skills despite the fact that they can improve attention control in older adults [20,21].

Recently, new techniques for cognitive stimulation and training have emerged, both from a preventive and rehabilitative perspective and among them, the use of musical stimuli as an integral part of the stimulation has become increasingly relevant as a research object over the past three decades [22,23].

As several scientific studies suggest, music has a wide range of effects on the brain [24,25,26], and musical learning has numerous benefits in terms of cognitive function development [27,28], even during aging [29,30]. Thus, the use of music in cognitive intervention represents a possibility with potential that is worthy of further investigation in the field of pathological aging [31].

Recent studies on the cognitive effects of music-based interventions in aging show statistically significant improvements in global cognitive functioning [32,33,34] but also in specific cognitive functions such as executive functions and episodic memory [35]. However, existing studies present research works with a high degree of heterogeneity in several characteristics, such as subject population [30,31], geographical location, techniques used, intervention contents, and context of application. This lack of uniformity in the results proposed among the studies does not allow for good generalizability of the effects, and therefore, exporting guidelines for interventions is not feasible [30,31]. Certainly, a first major distinction needs to be made about the type of intervention proposed; the literature is beginning to explore this aspect, but at the moment, there is not enough evidence to make well-founded assumptions.

For example, some studies focusing on the cognitive effects of listening to music found important results as early as the two decades between the late 1900s and 2000s. They reported benefits on autobiographical memory and performance on cognitive tests assessing improvements in both short- and long-term memory processes [36,37,38,39].

Conversely, more recently, in their meta-analysis, Fusar-Poli and colleagues [40] analyzed music therapy interventions by dividing them into two categories: active interventions (in which participants are also involved from a motor perspective) and receptive interventions (in which the main activity is listening to music). Discussion of the results obtained shows evidence of a beneficial effect of active MT on global cognition that does not emerge in music listening interventions. These findings guided the development of hypotheses about the key importance of cognitive abilities of aspects related to timing, sequencing, and the spatial organization of movements required in active music making. This issue has led to the specific study of the role of music and, in particular, rhythm as a cognitive rehabilitation technique in various neurodegenerative diseases characterized by progressive cognitive deterioration [41,42,43].

In this regard, the meta-analysis by Wang et al. [44] shows relevant data on the application of techniques based on the neurological music therapy (NMT) model, which includes a range of techniques aimed at treating cognitive, sensory, and motor dysfunctions resulting from neurological disorders [45]. However, Galińska [46], in this work, shows how NMT techniques could be applied, in principle, to neurogeriatric therapy as well. The results of the meta-analytic study conducted by Wang and colleagues [44] show how motor training based on rhythmic auditory stimulation (RASMT) can improve general cognitive status, memory, attention processes, and executive functions in people with cognitive impairment [44].

The technique engages a cortical network, including frontotemporal–parietal regions, through constant rhythmic stimulation that results in the activation of motor patterns. Compared to physical activity without music, cognitive function is improved here because it is possible that the involvement of this brain network through constant rhythmic stimulation promotes integration between the perceptual and cognitive systems by further coupling perceptual and motor events in older people with MCI [47].

These findings are supported by a series of other results shown in those studies that propose musical training to this population; actively making music with one’s body seems to have positive effects on memory, attention, and executive function [22,48,49].

From the aspects highlighted in this introduction, it is urgent to review the literature to further reflect on this variability, considering only those works focused on populations of patients with exclusively mild cognitive impairment, from MCI to mild dementia. We will analyze any type of proposed intervention that focuses on the use of music, including actual music therapy interventions (with a further distinction between active and receptive techniques), instrumental learning, singing groups, or other music-based interventions.

A scoping review was conducted to systematically analyze the research and identify gaps in the literature in an attempt to provide an evidence-based answer to the following questions. First, as a general question, we asked to what extent cognitive stimulation or training through music-based activities can actually contribute to the treatment of cognitive disorders in MCI and dementia (in stages where it is still possible to offer interventions requiring active patient participation, including only participants in the mild to moderate stages of dementia).

Second, we explored what type of music-based intervention is used and might be most effective for cognitive stimulation. Also, we asked whether the literature presents defined intervention protocols designed according to a specific therapeutic rationale. An additional element of interest concerns verification regarding the existence in the literature of music-based protocols designed to train specific cognitive functions according to the individual needs of people with deficits in one or more cognitive domains.

The review is interested in defining which assessment tools are most appropriate to gather evidence on the effectiveness of a treatment aimed at cognitive training; likewise, it is urgent to differentiate the results according to the extent of cognitive impairment in the sample to see if there are more suitable interventions for people in the preclinical stage than for individuals with dementia, even if in the early stage.

Therefore, a specific focus within the review is on analyzing the characteristics of interventions to focus on the components of a music-based intervention that is effective in treating people with MCI or early-stage dementia. The main objective is to promote the implementation of interventions adhering to good practices of cognitive stimulation or training.

## 2. Materials and Methods

As a methodology to present the current state of the art in music therapy research and to elaborate a critical analysis of the questions presented above, it was decided to adopt the scoping review methodology, which is considered suitable as it guarantees a precise mapping of a large set of evidence in a flexible and efficient way, in terms of the time–cost ratio, while maintaining a certain rigor and transparency. As a methodology, the scoping review also allows for the possibility of providing consultancy from experts who can further enrich the process with their experience, contributing to the critical synthesis of the results.

Throughout the review process, the PRISMA—ScR checklist was adopted as a point of reference and guidance [50], as well as the steps identified by Mak and Thomas [51], which included the following working phases after having well defined the research questions: identification of relevant studies, selection of studies to be included in the review, data extraction, and results presentation. The fundamental questions that guided the achievement of this scoping review are summarized below:

Can music-based interventions have any cognitive effects on older adults at early stages of cognitive impairment? To what extent, and which, are the most trained cognitive areas?

(1) Are there any differences in outcomes in participants with mild onset of cognitive decline (MCI) compared to participants with an already existing dementia diagnosis? What are the outcome differences between mild and moderate stages of dementia?
(2) Which types of music-based interventions are the most effective for cognitive stimulation, and how well defined are they in research articles?
(3) Do specific music-based protocols designed for the training of certain cognitive functions depending on the specific cognitive deficits of patients exist?
(4) Are there any assessment tools used in the majority of studies, and how far do they go in assessing the effects of an intervention on a specific cognitive function?

### 2.1. Search Strategy and Identification of Relevant Studies

As reference databases for the literature search and studies retrieval, the Web of Science and PubMed databases were used. The keywords included a single search and combination search through Boolean operators (AND, OR) of the words: ‘music’, ‘music-based intervention’, ‘music therapy’, ‘cognitive rehabilitation’, ‘cognitive stimulation’, ‘cognition’, ‘memory’, ‘executive functions’, ‘attention’, ‘cognitive impairment’, ‘Mild Cognitive Impairment’, ‘MCI’, ‘dementia’, ‘aging’, ‘elderly’.

In order to have a comprehensive and up-to-date look at the most recently proposed music-based interventions, we decided to limit the retrieval time to studies published in the last decade, i.e., from 2014 to 27 May 2024.

In the scope of the literature, searches were made for academic journals without any restriction on the type of study design but with the request to select only studies presented in the English language. The snowballing process was adopted, and reference list screening was achieved to identify additional relevant studies; as a matter of fact, we also carried out a screening of the bibliographical reference lists presented in the reviews, systematic reviews, and meta-analyses identified through the search string performed on the two databases.

### 2.2. Inclusion and Exclusion Criteria

Before proceeding with the process of selecting interesting studies for our research object, we established inclusion criteria and exclusion criteria that would help eliminate all studies not containing the necessary information to broaden the discussion on our research questions.

Therefore, studies were eligible for inclusion if they were original studies exploring the cognitive effects assessed after a music-based intervention and they respected the following requests specified by the PICOs criteria thus defined:

- Population: people with cognitive impairment related to aging, MCI, or dementia. Participants could be experiencing a range of disease stages, from MCI to moderate stages of dementia (the sample could include patients with Alzheimer’s disease-type dementia or any other defined type), but the severity should be clearly defined.
- Intervention: Music-based interventions (i.e., music therapy or other music-based activities): the idea was to select only interventions based on the use of music, characterized by group or individual sessions conducted by a professional figure according to a process idea, not single exposures to sound–musical stimuli.
- Comparison: the selection of interventions without the presence of a control group but with relevant results could be considered, keeping in mind, however, the greater reliability of studies comparing music-based treatments with a control group. In this regard, control groups treated with both standard care and other interventions based on other music-based or other non-pharmacological interventions were accepted in the selection process.
- Outcomes: test scores evaluating the global cognitive situation or performance tasks linked to specific cognitive areas or neuroimaging studies showing the effects of the intervention on a cortical activation level.
- Study design: given the nature of the review, no restrictions on study designs to be taken into consideration were placed. RCTs and CCTs were favored, but crossover, observational, cohort, and case studies could also be included in the review. Literature reviews and meta-analyses were additionally used as resources for selecting further relevant studies.

Furthermore, a number of other relevant aspects guided the exclusion process from the review. First of all, studies evaluating the cognitive effects of music-based interventions on patients with healthy aging or in a too-advanced stage of dementia were not taken into consideration, as well as patients with forms of cognitive deterioration secondary to another pathology (for example, Parkinson’s disease) or a traumatic and harmful event (such as in the case of a head injury or stroke).

As regards the type of intervention presented, all the studies evaluating the effects of a multi-component intervention, in which the music-based intervention occupied only a small part of the treatment or treatments in which music was used as background music to some other activity (i.e., interventions in which gymnastics programs are compared with the same interventions accompanied by music or cognitive stimulation interventions conducted with a musical background) were excluded from the analyses.

Finally, we also excluded all the studies that did not present a treatment but measured the performance of some cognitive or motor task after short musical playlist listening and studies aimed at evaluating differences in the cognitive reserve available to older adults with or without musical experience. All those studies that did not present actual results on cognitive tests but which only collected subjective data derived from the administration of scales to patients and caregivers regarding the perception of cognitive self-efficacy were also left out.

### 2.3. Study Selection

The results obtained from database searches were screened independently by two reviewers (FC, BA). After the removal of duplicates within the list of identified studies and an initial exclusion of irrelevant studies identified by reading the article titles, a screening of the abstracts was conducted, followed by a reading of the full texts of selected articles. All potentially relevant studies were assessed in detail according to the inclusion and exclusion criteria in order to definitely determine the studies meeting the established criteria. Both reviewers completed the process independently, discussing any concerns and inconsistencies after each step of the selection phase in order to resolve disagreements; afterwards, data were extracted and confirmed. Experts with specific knowledge of cognitive stimulation and cognitive training (psychologists) and clinical and research experience in dementia care were involved (FG, FB, and MM). Their contribution was asked to provide additional strength thanks to their competent point of view on the theoretical aspects and application implications regarding the topic of interest.

### 2.4. Data Extraction

Once the selection was achieved, the following step consisted of the extraction of relevant information for the analysis concerning a series of numerical data about demographic information on the population under analysis, such as average age, gender, nationality, and degree of cognitive impairment. All relevant information was then extracted regarding the types of intervention evaluated in the experimental condition(s) and in the control condition(s), and all the related details, such as frequencies, durations, contents, assessment methods of the cognitive outcomes collected, and main results deriving from the statistical analyses (all the primary and secondary cognitive outcomes were included, from the general cognitive status to attention, memory, language, and executive functions test scores). Furthermore, in some studies, the presence of results derived from the analysis conducted through neuroimaging techniques (in this case, functional magnetic resonance imaging and near infrared spectroscopy) was found, and the data of interest were extracted.

In the results and discussion sections, data are qualitatively taken into analysis, and implications for future music-based intervention design are discussed.

## 3. Results

### 3.1. Search Results

The search on the PubMed and Web of Science databases allowed the detection of a total of 849 studies on the topic, respectively, 426 and 423 articles. After extracting the complete list of studies on an Excel sheet, the removal of duplicates led to the exclusion of 177 studies. From the title and abstract reading, 604 irrelevant studies were excluded. Of these, 46 were reviews, systematic reviews, or meta-analyses; checking the bibliographical references of these studies allowed us to identify 12 further studies in compliance with the inclusion and exclusion criteria. In addition to these 12 studies, another 68 studies were then downloaded and screened as full text to assess the presence of the necessary information: 52 studies not meeting the criteria were removed. Of these, n = 11 for population issues (10 studies included healthy participants and 1 study participant with severe dementia only); n = 33 were excluded due to the inappropriateness of the proposed interventions (n = 7 studies fitness programs with music background; n = 13 interventions composed of a mix of different multisensory interventions; n = 13 interventions without music or a lack of intervention at all), n = 6 studies were not selected because cognitive outcomes were not provided, and n = 2 studies which were not retrievable in the English language.

**Figure 1:**
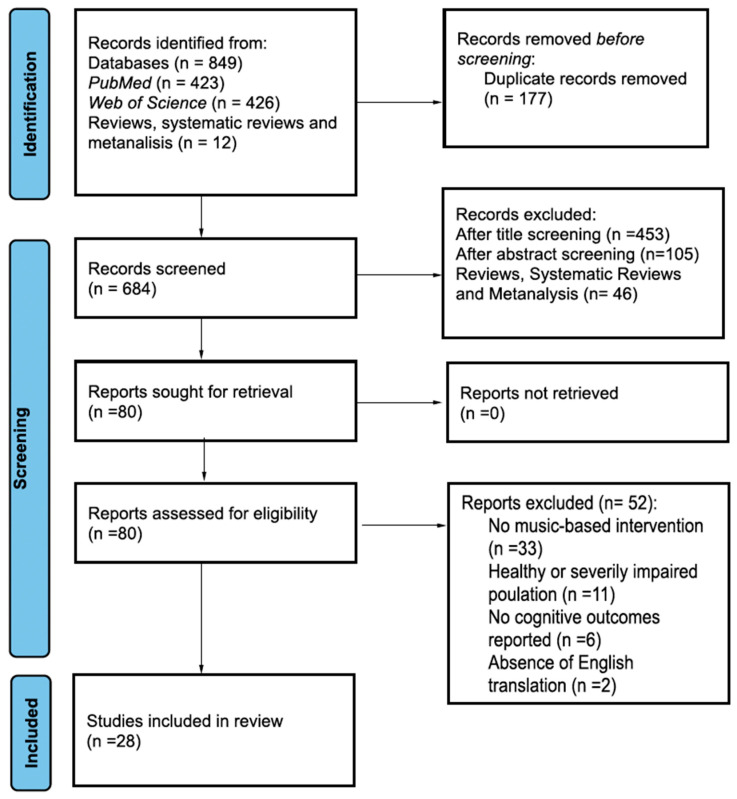
Flowchart of the search process.

At the end of the process, a scoping review was conducted on 28 studies [52,53,54,55,56,57,58,59,60,61,62,63,64,65,66,67,68,69,70,71,72,73,74,75,76,77,78,79]. Figure 1 presents the detailed selection process.

### 3.2. Studies Characteristics and Outcomes

Table 1 provides a summary of the relevant characteristics of the studies included in the review. The publication dates are in a time window that covers the last ten years, from 2014 to 2024, and the sample sizes range from 20 to 298 participants. The studies were conducted in various countries, including four different continents. N = 16 studies were conducted in Asia (n = 6 in China, n = 4 in Japan, n = 2 in Taiwan, n = 2 in Malaysia, n = 2 in Korea), n = 9 in Europe (n = 3 Italy, n = 2 Finland, n = 2 France, n = 2 Spain), n = 1 in Africa (Tunisia), n = 1 in South America (Brazil) and n = 1 in North America (USA).

Furthermore, 22 of the 28 studies considered were randomized controlled trials (RCT), adopting a between-groups research design with randomization. Four of the studies also present a comparison of effects between the experimental group and the control group, adopting a quasi-experimental design, as the division of participants into groups is not carried out through randomization techniques. Finally, the remaining two studies present a crossover design in which a single group participating in periods of activity alternating with periods of inactivity is evaluated longitudinally. The level of cognitive deterioration of the samples treated in the selected studies is rather heterogeneous, and in many cases, patients at different stages of decline were included in the same experiment. The list includes studies dealing only with patients with MCI (n = 5), studies that treat patients with mild dementia (n = 4) or moderate dementia (n = 1), works that include patients with MCI and mild dementia (n = 2), mild–moderate dementia (n = 12), and MCI and mild–moderate dementia (n = 2). Finally, two studies propose interventions carried out on a sample of older adults defined as fragile [58,70]; in the first case, the sample is made up of people who may have dementia but who also just need assistance, while in the second case, the population of interest is defined as at risk of developing dementia and the selected participants are people who perform below average in a series of cognitive tests (at least 5 out of 10 tests) or who complain of a subjective memory disorder. Furthermore, the study by Lyu et al. also includes participants with severe dementia in the sample but conducts the analysis stratified by level of impairment [66]. Similarly, the studies by Biasutti and Mangiacotti [52,53] and Maguire et al. [67] also include groups of people without full-blown cognitive impairment but treat them as a separate group in the analyses.

Regarding the content of the proposed music-based interventions, it is possible to divide the interventions into the following categories: active music-based interventions (n = 18), receptive music-based interventions (n = 8), and a mix of active and receptive music-based interventions (n = 4). Within the macro-category of active interventions, there was some heterogeneity of interventions, which needs to be further classified: of the 18 studies requiring the active participation of patients, n = 7 evaluate singing interventions, n = 5 belong to the field of music therapy interventions (n = 1 offers a relational improvisational music therapy intervention, n = 2 movement with music therapy, n = 1 applies neurological music therapy techniques, and n = 1 presents a song-based cognitive stimulation protocol developed from a therapeutic perspective), n = 2 present a cognitive training protocol with exercises designed to train specific cognitive functions and with a musical improvisation section; n = 1 consists of percussion training; n = 1 involves learning a song composed for the patient together with the patient; n = 1 is rhythmic training and n = 1 presents a mix of activities (including rhythmic, dance, and quiz activities).

Among the studies dealing with musical listening, some studies characterize their intervention as receptive music therapy (n = 2), others were based on listening to music preferred or familiar to the patients (n = 4), others use listening to perform reminiscence therapy (n = 1), and one study proposes listening to two single pieces recorded several times (n = 1). 

Another aspect to be taken into consideration is the characterization of the professional figure carrying out the treatment: in most cases, music-based interventions are led by trained music therapists (n = 16), whereas musical training related to the learning of singing or other musical instruments is conducted by music teachers or, in any case, by professional musicians (n = 7). In addition, other professionals in charge of the treatments are represented by music psychologists (n = 2), health professionals (i.e., nurses, n = 2), speech therapists (n = 1), self-administration with the help of a caregiver (n = 1), and, in one case, the professional figure is not specified, but it is clarified that the professional figure in charge of the treatment is an instructor duly trained in the application of the protocol (n = 1). Table 2 also provides details on the professional figure responsible for each intervention described.

In terms of the cognitive domains assessed in the studies reviewed (as shown in Table 3), the cognitive functions most commonly assessed as outcomes include measures of global cognition, memory processes, attention and speed processing, executive functions, reasoning, and verbal abilities. Among the measures used, there are also test batteries that present composite scores obtained from the measurement of different cognitive abilities but which provide global cognitive assessment scores. It can be highlighted that the Mini-Mental State Evaluation is the most widely used test of all (its score is present in 71.4% of the studies), followed by the TMT-A Attention Test (which assesses both attention and speed of processing), the Frontal Assessment Battery (a test that assesses global executive functioning), and the Verbal Fluency Test (for the assessment of language skills). Despite greater heterogeneity in the tests chosen to assess the mnemonic component, it can be noted how several studies focus on the assessment of these aspects, as well as with regard to the assessment of attention and executive functions (n = 13 studies assessing memory processes, n = 12 executive functions, n = 10 attention, n = 9 language, n = 4 reasoning).

Finally, of particular interest in terms of assessment methods are the studies that present data collected using neuroimaging techniques. In particular, the studies by Feng et al. [58], Satoh et al. [75], and Shimizu et al. [76]. who evaluate the effects of treatments using functional magnetic resonance imaging and biomarkers in the first two cases and NIRS technology in the other.

Regarding the main results of the studies, a significant improvement in global cognitive conditions, or at least in an aspect related to cognitive processes, can be observed in the majority of the studies, with the exception of the studies conducted by Giovagnoli et al. [60], Li et al. [64], Murabayashi et al. [70], Perez-Ros et al. [71], and Tang et al. [77]. Possible hypotheses regarding the reasons for the results obtained are proposed in the Discussion section.

However, as far as neuroimaging studies are concerned, both the studies by Satoh et al. [75] and Shimizu et al. [76] measure increases in the groups of participants who received music-based interventions, as highlighted in the first case by increased activity in the right angular gyrus and the left lingual gyrus, and in the second case by increased cerebral blood flow in the medial prefrontal cortex (mPFC). In contrast, the study by Feng et al. [56] found no differences in biomarkers (such as oxidative damage shown by liquid chromatography–mass spectrometry and immunosenescence measures intercepted in blood samples) between groups, with only gray matter (GM) volume, white matter (WM) fractional anisotropy (FA), and WM mean diffusivity (MD) showing small increases.

The following discussion is devoted to commenting on the results presented, with particular attention to the clinical implications of this research.

## 4. Discussion

This scoping review aimed to investigate the literature on the cognitive effects of music-based interventions on older adults with cognitive impairment. The results allow us to discuss the answers to the research questions that were relevant to us (see the Methods section). Regarding the most general question of whether there is evidence of cognitive improvements following experience with music-based treatments, the results of the reviewed studies show a good degree of consistency in emphasizing positive effects. The studies show improvement or at least maintenance (at the expense of deterioration reported in control conditions) of global cognitive status. The vast majority of studies here use a common assessment modality, the administration of the Mini-Mental State Evaluation test [80]. This is relevant to the generalization of the results, and rarely have studies found no significant improvements. This is the case of the studies by Moreira et al. [69], Li et al. [64], Perez et al. [71], and Tang et al. [77], whose statistics show no significant difference between the scores of the experimental and control groups and between the scores obtained in the pre- and post-intervention analyses. In any case, although the results are not significant, these studies have all shown that the overall cognitive status is maintained in the experimental group, while a deterioration is manifested in the control group. One hypothesis that could be made about these results would be related to the type of intervention proposed within these studies. As can be observed, two of the studies [64,71] present a standard music listening intervention proposed to all participants in the experiment: in the first case, the program proposes to listen to the same two recorded songs repeatedly in each session to reach a duration of 30 min; in the second case, a playlist of music that patients enjoy and are familiar with is created with the support of patients and caregivers, but then used in the same way for all participants. Furthermore, in the case of this work, both experimental and control groups receive occupational therapy, which could explain the lack of additional effects in the experimental group. Instead, in the studies by Moreira et al. [69] and Tang et al. [77], the interventions propose a mix of activities that include active musical participation (patients are asked to engage in learning musical instruments and music) and also listening to music. However, in the case of Moreira’s study, the proposed protocol is based on the application of music therapy techniques (NMT), which, in any case, have allowed us to detect significant improvements in the cognitive area of episodic memory. An important question that arises, therefore, concerns the choice of assessment tools to be preferred in the evaluation of a music-based intervention. In terms of cognitive assessment, neuropsychological test batteries have been the most widely used tool to date and are the most suitable at detecting tangible cognitive improvements (although in only three cases [58,75,76] were the results enriched by the use of neuroimaging techniques, which are undoubtedly more complicated in terms of implementation). However, with regard to assessment tools for single aspects of specific cognitive domains, there is greater heterogeneity and personalization in the choice of tools to be adopted. For example, in the cognitive domain of memory, one of the domains most affected by cognitive decline, there are no common tools used in the studies, and even the results obtained from the administration do not show a certain homogeneity. In any case, many of the studies evaluating some mnemonic components show significant benefits from the point of view of mnemonic processes [56,57,59,66,68,69,74,75]. Regarding the cognitive areas that concern attention, executive functions, and language, other areas that acquire a particular relevance in the evaluation of cognitive deterioration, some assessment tools are adopted in greater percentages by studies; for attention, the most used test is the Trial Making Test (TMT) [81]; for executive functions, the Frontal Assessment Battery (FAB) is used [82]; and for language, it is the Verbal Fluency Test (VFT) [83]. Regarding the results obtained from the administration of the FAB and the VFT, there is good agreement in the significant positive effects found, while regarding the TMT, the results are divided into a part where a positive effect is found and a part where no significant improvement is found. Therefore, the age and cognitive conditions of the sample may be a rather important factor in the choice of tests, especially in the case of this last test, which measures variations in information processing speed that are difficult to detect in the elderly population. Clearly, understanding which tests may be the most valid in the evaluation of a music-based intervention remains an open issue, and it is hoped that future studies will address the issue of assessment in detail.

Another aspect of interest that is worthy of consideration is the presence or absence of differences in outcomes between participants with mild cognitive impairment (MCI) and those with a diagnosis of dementia. Unfortunately, most of the studies reviewed present clinical populations that often include groups of patients with MCI, mild dementia, or moderate dementia at the same time, without specifying the type of dementia considered and without making distinctions in the analyses of the outcomes collected before and after the intervention. All the studies that did not find statistically significant results used a rather heterogeneous population as the reference population, which may, in fact, have very different pathological conditions. An example is the study by Murayabashi et al. [68], which identifies the study population as a population of older adults in fragile conditions (thus including, in addition to patients with full-blown dementia, also people with a need for assistance resulting from a loss of autonomy from a physical or motor point of view, not necessarily cognitive). Obviously, hypothesizing about the stages at which music intervention might be most valuable is made more difficult by this aspect. An exception is the methodological rigor of the studies by Biasutti et al. [52,53], Lyu et al. [66], and Sarkamo et al. [73], who, in their papers, arranged analyses for population subgroups. Biasutti et al. [52,53], for example, have distinguished two groups of interest according to a threshold represented by the baseline score on the MMSE (a subsample characterized by overtly poor scores and another composed of all those individuals with scores tending toward or within the norm). The effects on global cognitive status observed in these cases are statistically significant for the population with deficient scores, i.e., in a state of overt dementia, while no improvement is observed in the group with normal global cognition. This result shows that cognitive stimulation is particularly effective in cases where cognitive decline is evident. Sarkamo’s study [73], from the results obtained by analyzing subgroups of patients with mild dementia and patients with moderate dementia, shows how specifically listening to music correlates with improvements in global cognitive level, which are greater in the subgroup of patients in more moderate phases compared to the initial ones. Lyu’s study [66], on the other hand, shows how the intervention is more effective in patients with an initial level of cognitive decline than in those with more fundamental impairment on certain language and memory tests by analyzing two subgroups, one with mild dementia and the other with moderate dementia. These studies underscore the importance of intervening with music-based interventions throughout the aging process, with the possibility of observing benefits from the point of view of global cognitive stimulation in more advanced stages (also through musical listening techniques) and training of specific cognitive functions in the early stages of cognitive decline (through active participation in engaging activities). It is clear that in order to generalize about the applicability of specific intervention protocols, there is an increasing need to subdivide the population in studies in order to obtain a greater amount of evidence.

Another relevant aspect that should be noted is the preponderance of active control groups in the studies analyzed. In fact, 16 of the studies compare the effect of interventions based on the use of music with several other types of stimulation, such as physical activities like dance or gymnastics, physiotherapy, occupational therapy, pharmacological therapy, educational or socialization interventions, reading stimulation or traditional cognitive training, or other activities related to visual art forms such as painting. In most cases, however, an improvement or at least comparable effects to the other interventions were observed, suggesting that the therapeutic effect of music may have its own specificity.

A final question to be addressed concerns the verification of the existence of some interventions with music in the literature. These interventions present specific intervention protocols adapted to the cognitive function to be trained. The studies presented, published in the last decade, all contain a more or less developed section in which the content of the proposed interventions is briefly presented. Although this is a step forward in the definition of what is done, there is often a lack of reference to the reasons why certain activities are proposed. In addition, some of the studies lack a clear definition of the stimulation parameters, that is, the details regarding the time, frequency, duration, and, above all, the rationale of each activity that go beyond a generic description of what is done. However, among the works, some protocols designed for training or cognitive stimulation stand out and deserve to be mentioned in more detail [53,54,59,62,69]. The studies cited above, based on recent findings in the neuroscience of music and recognized models of music therapy, such as NMT, present protocols with an excellent level of definition. In particular, the description of the protocols indicates the presence of musical exercises specifically designed to train a particular cognitive goal. For example, in the case of Chen et al. [54], a protocol based on the need to train attention control is developed; starting from a non-musical exercise, the authors created a series of musical exercises that train this cognitive function by requiring the performance of a motor task and a cognitive task at the same time. In the case of Han et al. [62], instead, the Song-based Cognitive Stimulation Therapy protocol was created, consisting of a digital program of musical stimuli selected by a trained music therapist with the aim of facilitating the stimulation of cognitive processes defined through music. In the case of the studies by Biasutti et al. [53] and Moreira et al. [69], the goals of stimulation are clearly defined in their protocols in terms of cognitive functions trained through specific exercises, such as scat improvisation [53] and defined temporal planning of the activities to be performed and the processes to be trained [69]. Finally, the study by Fraile et al. [59] proposes a very interesting protocol in which an effective song is created for the patient, using very dense and detailed language, and then this song is learned during sessions to stimulate both autobiographical memory and language skills (the protocol is actually carried out by an expert speech therapist). The existence of these protocols gives hope that increasingly precise levels of definition will be achieved in studies presenting music therapy interventions.

### 4.1. Implications for Practice

The analysis of the studies in this review leads to the following implications and recommendations from the perspective of cognitive stimulation best practices in treating cognitive impairment, which are applicable to music-based interventions.

The literature on cognitive interventions for patients with MCI or dementia generally agrees that treatment should be patient tailored. Consequently, their design should take into account disease severity, general health status, cognitive reserve, living environment, and the quality and level of caregiver support. The choice of intervention depends on the severity of the disease and the primary goals at that stage. The main application advice is to focus on the design of protocols that take into account whether the intervention is intended to be cognitive stimulation or cognitive training, depending on the stage of deterioration the person is in.

In addition, the cognitive load of the intervention must be calibrated to the patient’s residual capacities. In order to monitor the difficulty, it is useful to modify the exercises sequentially, for example, by increasing the number of stimuli the person has to process in a single session and subsequently reducing the presentation time of the stimuli. In this way, the level of difficulty must be set gradually and incrementally, keeping it at a threshold, i.e., at the right limit with respect to the person’s abilities. Activities must be cognitively stimulating and tuned in a range of functional complexity to trigger plasticity processes. Furthermore, the patient must be actively involved in each session, sharing objectives and explaining what is being done. The treatment should include frequent, well-timed, and stable meetings over time, e.g., every day at the same time and location for a given number of weeks, so as to encourage the acquisition of a time routine for carrying out the activities. It is also advisable to conduct the session in the morning hours, leaving the rest of the day for activities that require less intense and demanding participation. Moreover, the literature suggests that a CST program should include a minimum of 14 structured group sessions, biweekly, of approximately 45 min each [84]. Additionally, the presence of professionals with specific training to deliver the treatment is required. In fact, the only studies in which there is a greater likelihood of finding no effect of CST on general cognitive function are those in which the intervention is individualized and delivered at home, as shown in the analysis of the intervention by Li et al. [64]. This trend also suggests the importance of the group context as a source of stimulation for patients with dementia [85]. Especially from a psychological and cognitive point of view, the group context represents a fundamental source of stimulation and leads to multiple interconnected positive effects, such as increased patient engagement in cognitive treatment activities, which, in turn, may promote cognitive stimulation. On a qualitative level, patients may perceive improvements in memory and attention [86]. One relevant aspect, however, concerns the fact that the group should be fairly homogeneous, i.e., composed of patients at the same stage of disease severity in order to meet the individual and group rehabilitation needs.

Finally, with respect to the methods of cognitive assessment of treatment outcomes, a deeper insight is needed regarding the use of the most commonly administered test in the various studies, the MMSE. As the MMSE has a highly verbal and less visuo-spatial content, it would be recommended to combine other global screening tests, such as the Montreal Cognitive Assessment (MoCA) in the assessment, which allows a wider spectrum of cognitive domains to be investigated and contains more sensitive tests of attentional and frontal functions. Moreover, in dementia, cognitive deficits are associated with changes in emotional state and behavioral disturbances, so an evaluation of the effects of cognitive stimulation interventions with music on the emotional–behavioral domain (multidimensional assessment) is also needed.

### 4.2. Limits and Future Research

Evidence to date confirms the importance of cognitive interventions in the clinical toolkit to help people with dementia cope with some of the everyday barriers caused by cognitive and functional difficulties. Through the analysis of the studies presented in this review, the same statement can be extended to music-based interventions. However, research has a number of limitations due to confusion over which interventions are considered cognitive and which are not. For example, many approaches, including those based on music, include interventions that use a mix of techniques, reality orientation therapy, reminiscence, and socialization activities, but often without specific awareness of the functions being trained. In addition, there are a variety of settings and ways of implementing interventions with different methodologies and tools for evaluating outcomes. This leads to a degree of difficulty in determining whether or not specific cognitive interventions are effective in dementia. Therefore, there is a need to establish standard criteria for defining levels of disease severity, guidelines for designing patient-centered interventions, and follow-up to examine the significance of effects over time [87].

Compensatory neuroplasticity processes are particularly active during the silent phase of dementia, and they could be stimulated to postpone the cognitive decline that leads to the more severe symptoms that define dementia [88]. The existing evidence basis is promising, but some questions remain to be answered in further research. First of all, a better understanding is needed of the critical period for which intervention with training or stimulation with music is required. The pathological cascade leading to dementia, which is likely to begin many years before the diagnosis of dementia, suggests that these programs are likely to have the greatest effect if provided early in the MCI phase or perhaps even before this phase. In addition, the effectiveness of cognitive training may benefit from a better characterization of individuals with MCI who will progress to dementia. Several studies have shown that cognitive tests can discriminate between those who will later progress to dementia and those who will remain stable [89].

## 5. Conclusions

The results of this review are encouraging and suggest the introduction of music-based interventions with stimulation/prevention purposes as complementary approaches to usual cognitive treatments. The use of standardized protocols from active and/or receptive approaches (also considering the ‘dose/effect’ ratio) is therefore suggested. In particular, the need for interventions aimed not only at global cognitive stimulation but also concerning specific cognitive functions is advisable. In comparison to the main music therapy literature [90], music-based interventions are also recommended in the early stages of dementia, in MCI, and, in a preventive sense, in healthy older adults. From a research point of view, it will be important, in addition to a strong methodological approach and the definition/description of the intervention protocols, to accurately define the target population and related cognitive disorders. In addition to this, an established assessment evaluating the results at the clinical and neuroscientific level (possible changes in brain plasticity and connectivity) is also needed.

## Figures and Tables

**Table 1:** Characteristics of included studies.

Study and Year [Ref.]	Study Design	Country	No. of Participants	Mean Age	Female	Severity of Cognitive Impairment	Type of Music-Based Intervention	Control Type	Intervention Period	Intervention Frequency	Intervention Duration	Cognitive Outcomes	Cognitive Results
Biasutti and Mangiacotti 2021 [52]	^1^ RCT	Italy	45	84.5	29/64.4%	^2^ MCI and mild dementia	Music training (improvisation exercises)	Gymnastic activity	6 weeks	2 per week	70 min	^3^ MMSE	Improvement
Biasutti and Mangiacotti 2018 [53]	RCT	Italy	35	83.57	23/65.7%	MCI and mild dementia	Music training (improvisation exercises)	Gymnastic activity (45 min)	6 weeks	2 per week	70 min	MMSE, ^4^ VFT, ^5^ TMT-A, attentional matrices, ^6^ CDT	Improvement in MMSE, VFT, CDT
Chen 2018 [54]	RCT	Taiwan	28	77.3	14/50%	Mild–moderate dementia	Musical dual-task training (^7^ NMT)	Dual-task training	8 weeks	1 per week	60 min	TMT-A	Improvement
Chéour 2023 [55]	RCT	Tunisia	28	72.8	12/42.85%	Mild ^8^ AD	Music listening	Physical rehabilitation PR + ML, control	4 months	3 times per week	60 min	MMSE, ^9^ ADAS- Cog	Improvement (in ML, PR, and PR + ML)
Cheung 2018 [56]	RCT	China	165	85.3	125/75.75%	Moderate dementia	Movement music Therapy (MM)	Music listening (ML), Social activity	6 weeks	2 per week	40 min	MMSE; ^10^ FOME; Mod-VFT; Digit Span	Improvement (MM, ML) in MMSE; memory storage and recall; VFT improvement in MM group
Doi 2017 [57]	RCT	Japan	201	76	104/51.7%	MCI	Playing instrument (percussions)	Dance, control	10 months	1 per week	60 min	MMSE, TMT-A, TMT-B, story, and word memory	MMSE improvement (music group); improvement memory recall (dance group only)
Feng 2020 [58]	RCT	Malaysia	93	70	73/78.5%	At risk of dementia (early cognitive impairment in 5/10 cognitive tests)	Choral singing	Health education	2 years	1 per week	60 min	^11^ CCTS, ^12^ SM-MMSE ^13^ MRI, oxidative damage/ immunosenescence	Improvement without intergroup difference; no difference in bio- markers
Fraile 2019 [59]	^14^ CCT	France	12	83.83	7/60%	Mild–moderate AD	Learning an individualized song (lyrics writing)	Control	5 weeks + 5 weeks (Waitlist)	2 per week	20 min	Cued autobiographical recall, phonological, and semantic fluency; ^15^ EFCL (verbal, memory, executive process)	Improvement in autobio- graphical memory retrieval and general cognitive abilities
Giovagnoli 2017 [60]	RCT	Italy	39	73.6	24/61.5%	Mild–moderate AD	Active music therapy	Active control (cognitive training CT, neuro- education NE)	12 weeks	2 per week	45 min	TMT, ^16^ DSST	No significant changes; clinically significant improvement rates = CT: (62%) AMT: (8%) NE: (none)
Gómez- Gallego 2021 [61]	Quasi- experi- mental design (Randomization of nursing homes)	Spain	90	80.9	55/61.1%	Mild–moderate dementia (probable AD)	Active music intervention (AMI); receptive music Intervention (RMI)	Control pharmacological therapy, cognitive stimulation	12 weeks	2 per week	45 min	MMSE	Improve- ment in AMI (higher than in RMI)
Han 2020 [62]	RCT, pilot	Korea	24	73.12	11/45.8%	MCI	Song-based cognitive stimulation protocol	Control	10 weeks	2 per week	60 min	MMSE-DS ^17^ MoCA-K	Improvement
Kim and Kang 2021 [63]	RCT, pilot	South Korea	49/40	81.6	31/77.5%	Mild–moderate dementia	Active music intervention (rhythmic exercises)	Control (usual care)	12 weeks	2 per week	50 min	MMSE- Korean version	Improvement
Li 2015 [64]	Quasi- experi- mental trial design	Taiwan	41	78.75	28/68.29%	Mild AD	Music listening (2 different pieces)	Control	6 months	2 daily	30 min	^18^ CASI, CASI- estimated MMSE	No improvement (less decreased score than controls)
Liu 2024 [65]	RCT	China	24	69.45	12/50%	Mild–moderate Dementia	Group music therapy (activities change across sessions)	Control (usual care)	5 months	1 per week	40 min	MMSE	Improved scores (without statistics)
Lyu 2018 [66]	RCT	China	298	69.7	173/58.1%	^10^ AD (mild, moderate, severe)	Singing (S)	Reading (R), control	12 weeks	2 per week (2 per day)	30–40 min	VFT, ^19^ AVLT, MMSE	Improvement in VFT (S and R groups) and in immediate recall (singing)
Maguire 2015 [67]	No rando- mization (voluntary partici- pation)	USA	45	70–99	38/85%	MCI and mild–moderate dementia	Singing (vocal training)	Music listening	16 weeks	3 times per week	50 min	MMSE, CDT	Improvement in MMSE (both groups, larger effect in singing group)
Mahendran 2018 [68]	RCT, pilot	Malaysia	68	71.1	38/55.9%	MCI	Music listening ML (reminiscence)	Art therapy, control	12 weeks	1 per week	65 min	AVLT	Improvement in ML group
Moreira 2023 [69]	RCT	Brazil	43	76.49	39/91%	MCI and mild–moderate dementia	Neurologic music therapy	Control	6 weeks	2 per week	30–40 min	^20^ WAIS-III Digit subtest; Corsi block-tapping test; ^21^ FMT; ^22^ SASMET; CDT; MMSE	Improvement in episodic memory tests only
Murabayashi 2019 [70]	CCT	Japan	115	81.3	109/93.6%	Frail elderlies (with dementia or other care needs)	Music listening and singing	Control	12 weeks +12 weeks (Waitlist)	1 per week	45–50 min	VFT: ^23^ YKSST	No significant difference observed either in the period- effect or treatment for cognition
Perez-Ros 2019 [71]	RCT	Spain	119	80.52	61/51.26%	Mild–moderate dementia	Preferred music listening + occupational therapy	Occupatio- nal therapy	8 weeks	5 days per week	60 min	MMSE	Mainte- nance (worse- ning in controls)
Pongan 2017 [72]	RCT	France	59	79.5	39/66.1%	Mild AD	Singing	Paint	12 weeks	1 per week	120 min	TMT-A, ^24^ FAB	Improvement (digit, inhibitory processes) in both groups; singing group: verbal memory stable
Särkämö 2016 [73]	RCT	Finland	89	78.3	55/51.4%	Mild–moderate dementia	Singing groups, music listening groups (ML)	Standard Care	10 weeks	1 per week	90 min	MMSE, ^25^ WMS-III FAB, WAIS-III, TMT-A, ^26^ BNT; ^27^ WAB	Singing: improvement in working memory; maintenance: executive function, orientation (mild dementia); ML-supported general cognition, working memory (moderate dementia)
Särkämö 2014 [74]	RCT	Finland	89	78.8	60/67.4%	Mild–moderate dementia	Singing groups, music listening groups	Standard Care	10 weeks	1 per week	90 min	MMSE, WMS-III, FAB, WAIS-III, TMT-A, BNT, WAB	Experimental groups: improvement orientation, remote episodic memory; attention, executive function, general cognition slightly improved; effect of singing also in short-term and working memory
Satoh 2015 [75]	CT	Japan	20	77.55	14/70%	Mild–moderate AD	Singing training	Control	6 months	1 per week	60 min	MMSE, ^28^ RCPM, ^29^ RBMT, FAB, fMRI assessment	Time for RCPM completion significant reduction; increased activity in the right angular gyrus and the left lingual gyrus (fMRI)
Shimizu 2018 [76]	RCT	Japan	45	74.64	38/84.4%	MCI	Movement music therapy (MMT)	Gymnastic activity	12 weeks	1 per week	65 min	FAB, ^30^ CBF with ^31^ NIRS	Improvement (FAB) in MMT; significant increase in CBF
Tang 2018 [77]	RCT	China	77	75.88	38/49.4%	Mild–moderate AD	Listening, singing, and playing instruments	Control	12 weeks	3 times per week	50 min	MMSE	Mainte- nance (decrease in controls)
Wang 2018 [78]	RCT	China	60	69.75	38/63.3%	Mild AD	Listening and singing familiar songs (+pharmacolo- gical therapy)	Pharmacological therapy only	12 weeks	3 times per day	30–50 min	MMSE, MoCA	Improvement (MMSE); listening group. MoCA significant increase (both groups)
Xue 2023 [79]	RCT	China	80	74.93	62/77.5%	MCI	Receptive music therapy (RMT)	Standard Care	8 weeks	4 times per week	20 min (+time for the last two session phases)	MoCA	Improvement in RMT (especially memory, attention, abstraction)

^1^ RCT = randomized controlled trial; ^2^ MCI = mild cognitive impairment; ^3^ MMSE = Mini-Mental State Evaluation test;^4^ VFT = Verbal Fluency Test; ^5^ TMT-A/B = Trail Making Test; ^6^ CDT = Clock Design Test; ^7^ NMT = neurologic music therapy; ^8^ AD = Alzheimer’s disease; ^9^ ADAS-Cog = Alzheimer’s Disease Assessment Scale—Cognitive; ^10^ FOME = Fuld’s Object Memory Evaluation; ^11^ CCTS = composite cognitive test score; ^12^ SM-MMSE = Singapore Modified MMSE; ^13^ MRI/fMRI = (functional)Magnetic Resonance Imaging; ^14^ CCT = Controlled Crossover Trial; ^15^ EFCL = Language Cognitive Functions Assessment; ^16^ DSST = Digit Symbol Substitution test; ^17^ MOCA-K = Montreal Cognitive Assessment (Korean); ^18^ CASI = Cognitive Abilities Screening Instrument; ^19^ AVLT = Auditory Verbal Learning Test; ^20^ WAIS = Wechsler Adult Intelligence Scale; ^21^ FMT = Figure Memory Test; ^22^ SASMET = Musical Autobiographical test; ^23^ YKSST = Yamaguchi Kanji Symbol Substitution Test; ^24^ FAB = Frontal Assessment Battery; ^25^ WMS = Wechsler Memory Scale III; ^26^ BNT = Boston Naming Test; ^27^ WAB = Western Aphasia Battery; ^28^ RCPM = Raven’s Colored Progressive Matrices; ^29^ RBMT = (Rivermead Behavioral Memory Test; ^30^ CBF = cerebral blood flow; ^31^ NIRS = functional near-infrared spectroscope. From the analysis of these studies, it is interesting to see how the group dimension is favored over the management of individual interventions (n = 23 group interventions and n = 5 individual interventions). In some studies, the group size is not specified, in others, it is rather large (as in the case of the studies by Tang et al. [77], Lyu et al. [66], Gomez-Gallego et al. [61], in which the size per group is greater than 6 individuals). Details of each study are given in Table 2.

**Table 2:** Details on music-based intervention providers and type of individual/group intervention.

Study and Year [Ref.]	Music Intervention Provider	Group or Individual Intervention
Biasutti and Mangiacotti 2021 [52]	Music psychologist	Group
Biasutti and Mangiacotti 2018 [53]	Music psychologist	Group
Chen 2018 [54]	Music therapist	Individual
Chéour 2023 [55]	Music therapist	Group (with caregivers)
Cheung 2018 [56]	Health care professional	Group (4–6 people)
Doi 2017 [57]	Music teacher	Group
Feng 2020 [58]	Professional musicians	Group
Fraile 2019 [59]	Language and speech therapist trainees	Individual
Giovagnoli 2017 [60]	Music therapist	Individual
Gómez-Gallego 2021 [61]	Art therapists (specialized in music therapy)	Group (6–9 people)
Han 2020 [62]	Music therapist (and neurologist)	Group
Kim and Kang 2021 [63]	Music therapist	Group
Li 2015 [64]	Self-administration (with the help of caregivers)	Individual
Liu 2024 [65]	Music therapist	Group
Lyu 2018 [66]	Music therapist	Group (6 people)
Maguire 2015 [67]	Vocal teacher (+DVD instructor)	Group
Mahendran 2018 [68]	Art therapist (specialized in music)	Group
Moreira 2023 [69]	Music therapist (and psychologist)	Individual (with caregiver presence)
Murabayashi 2019 [70]	Music therapist	Group
Perez-Ros 2019 [71]	Nurses	Group
Pongan 2017 [72]	Choir conductor (with a psychologist)	Group
Särkämö 2016 [73]	Music therapist and Vocal teacher	Group (10 people = 5 caregiver-patient dyads)
Särkämö 2014 [74]	Music therapist and Vocal teacher	Group (10 people = 5 caregiver-patient dyads)
Satoh 2015 [75]	Vocal teacher (+a pianist)	Group
Shimizu 2018 [76]	Instructor (trained in protocol administration)	Group
Tang 2018 [77]	Music therapist	Group (9 people)
Wang 2018 [78]	Music therapist	Group
Xue 2023 [79]	Music therapist	Group

**Table 3:** Cognitive area assessed, relative neuropsychological tests.

Cognitive Area	Cognitive Measures	No. of Studies
Global Cognition (or composite score cognitive test)	MMSE	20
	MoCA	3
	AVLT	2
	CASI	2
	ADAS-Cog	1
	CCTS	1
Memory	AVLT	2
	Digit Span	2
	WMS	2
	Corsi block-tapping test	1
	Cued autobiographical recall	1
	FMT	1
	FOME	1
	RBMT	1
	SASMET	1
	Story and word memory	1
Attention (Speed processing)	TMT-A	7
	Attentional matrices	1
	DSST	1
	YKSST	1
Executive Functions	FAB	5
	CDT	3
	Digit Span Backward	2
	TMT-B	2
Reasoning	WAIS	3
	RCPM	1
Language	VFT	4
	BNT	2
	EFCL	1
	Phonological and semantic fluency	1
	WAB	1
Neuroimaging techniques	fMRI (cerebral activity, immunosenescence)	2
	CBF with NIRS	1

MMSE = Mini-Mental State Evaluation test; VFT = Verbal Fluency Test; TMT-A/B = Trail Making Test; CDT = Clock Design Test; ADAS-Cog = Alzheimer’s Disease Assessment Scale—Cognitive; FOME = Fuld’s Object Memory Evaluation; CCTS = composite cognitive test score; MRI/fMRI = (functional) magnetic resonance imaging; EFCL = Language Cognitive Functions Assessment; DSST = Digit Symbol Substitution test; MOCA-K = Montreal Cognitive Assessment (Korean); CASI = Cognitive Abilities Screening Instrument; AVLT = Auditory Verbal Learning Test; WAIS = Wechsler Adult Intelligence Scale; FMT = Figure Memory Test; SASMET = Musical Autobiographical test; YKSST = Yamaguchi Kanji Symbol Substitution Test; FAB = Frontal Assessment Battery; WMS = Wechsler Memory Scale III; BNT = Boston Naming Test; WAB = Western Aphasia Battery; RCPM = Raven’s Colored Progressive Matrices; RBMT = Rivermead Behavioral Memory Test; CBF = cerebral blood flow; NIRS = functional near-infrared spectroscope.

## Data Availability

No new data were created or analyzed in this study.
